# An update on the use of sphingosine 1-phosphate receptor modulators for the treatment of relapsing multiple sclerosis

**DOI:** 10.1080/14656566.2023.2178898

**Published:** 2023-03-22

**Authors:** Laura Dumitrescu, Athanasios Papathanasiou, Catalina Coclitu, Afagh Garjani, Nikos Evangelou, Cris S. Constantinescu, Bogdan Ovidiu Popescu, Radu Tanasescu

**Affiliations:** a Department of Clinical Neurosciences, University of Medicine and Pharmacy Carol Davila, Bucharest, Romania; b Department of Neurology, Colentina Clinical Hospital, Bucharest, Romania; c Department of Neurology, Queen’s Medical Centre, Nottingham University Hospitals, Nottingham, UK; d Department of Multiple Sclerosis and Neuroimmunology, CHU Grenoble, Grenoble, France; e Academic Clinical Neurology, Mental Health and Clinical Neurosciences Academic Unit, School of Medicine, University of Nottingham, Nottingham, UK; f Department of Neurology, Cooper Neurological Institute, Camden, NJ, USA

**Keywords:** Fingolimod, multiple sclerosis, ozanimod, ponesimod, sphingosine 1-phosphate receptor modulators, siponimod

## Abstract

**Introduction:**

Multiple sclerosis (MS) is an immune-mediated disorder of the CNS manifested by recurrent attacks of neurological symptoms (related to focal inflammation) and gradual disability accrual (related to progressive neurodegeneration and neuroinflammation). Sphingosine-1-phosphate-receptor (S1PR) modulators are a class of oral disease-modifying therapies (DMTs) for relapsing MS. The first S1PR modulator developed and approved for MS was fingolimod, followed by siponimod, ozanimod, and ponesimod. All are S1P analogues with different S1PR-subtype selectivity. They restrain the S1P-dependent lymphocyte egress from lymph nodes by binding the lymphocytic S1P-subtype-1-receptor. Depending on their pharmacodynamics and pharmacokinetics, they can also interfere with other biological functions.

**Areas covered:**

Our narrative review covers the PubMed English literature on S1PR modulators in MS until August 2022. We discuss their pharmacology, efficacy, safety profile, and risk management recommendations based on the results of phase II and III clinical trials. We briefly address their impact on the risk of infections and vaccines efficacy.

**Expert opinion:**

S1PR modulators decrease relapse rate and may modestly delay disease progression in people with relapsing MS. Aside their established benefit, their place and timing within the long-term DMT strategy in MS, as well as their immunological effects in the new and evolving context of the post-COVID-19 pandemic and vaccination campaigns warrant further study.

## 1. Introduction

Multiple sclerosis (MS) is a chronic immune-mediated disorder of the central nervous system (CNS) [1]. It affects around 2.8 million people worldwide, females twice as likely as males [2]. The estimated global prevalence is 35.9 per 100,000 people, ranging up to more than 200 per 100,000 people in Northern Europe, Germany, Italy, and North America [2]. In the UK, the prevalence of MS is 290 per 100,000 in Scotland, and 190 and 176 per 10,000 in England and Wales, respectively [3]. Despite the development of moderate and high efficacy disease-modifying therapies (DMTs), curative treatments are not available and MS remains a leading cause of neurological disability [4]. Its typical clinical onset is in young adulthood, which contributes to the high socio-economic burden [4].

The etiopathogenesis of MS is complex and highly heterogenous with both genetic and environmental factors being involved [1]. Pathologically, MS is characterized by chronic widespread low-grade neuroinflammatory changes and recurrent attacks (also known as relapses) of focal inflammatory demyelinating lesions, with variable degrees of clinical expression and recovery, followed by periods of remission [1]. Progressive neurodegeneration is triggered early in the course of the disease, subclinical at first, then resulting in gradual disability accrual unrelated to the relapses [5].

The main phenotypes of MS are relapsing-remitting (RRMS), secondary progressive (SPMS), and primary progressive (PPMS) [1,6]. The relapsing-remitting phenotype is present at onset in about 90% of the cases and is usually followed by a secondary progressive phase (i.e. SPMS) consisting of gradual disability accrual, with (i.e. active SPMS) or without overlapping relapses [1]. Clinically isolated syndrome (CIS), defined as the first clinical attack most likely related to MS but not yet fulfilling MS diagnostic criteria is another potential presentation [1,7]. Around 1 in 8 people with MS are diagnosed with PPMS. In PPMS, the symptoms gradually worsen and accumulate over time, in the absence of overt clinical relapses, but overt clinical relapses may sometimes occur (in 3% of cases i.e. progressive-relapsing MS).

The approved DMTs for MS are oral, self-injectables and infusible immunomodulators and immunosuppressants [8–10]. These drugs act on the inflammatory component of the disease, preventing the formation of new CNS lesions and the reactivation or enlargement of preexistent lesions, and reducing the annualized relapse rate with moderate-to-high efficacy – sphingosine 1-phosphate (S1P) receptor (S1PR) modulators belonging to the latter group, along monoclonal antibodies and cladribine [8–10]. The impact of current DMTs on slowing down the neurodegenerative component and reducing MS progression are minor or debatable, while drugs with clinically proven neuroprotective or neuroregenerative/promyelinating effects are not available yet. In view of the above, early control of disease activity remains the best available strategy for achieving good long-term outcomes in people with MS. The latest guidelines recommend that people with relapsing forms of MS, including CIS, RRMS, and active SPMS, start treatment with one of the available DMTs as early as possible, higher disease activity justifying the choice of a drug that is more efficacious but poses greater safety concerns. Current treatment guidelines in Norway recommend that newly diagnosed RRMS patients are commenced directly on a high-efficacy DMT, unless that there are specific reasons not to do so [11]. Concurrently, the presence of disease activity despite the use of a DMT mandates the switch to another, preferably more efficacious, drug [8–10]. The best strategy (escalation vs induction) in the DMT sequence is still debated, and clinical studies addressing this are underway (clinicaltrials.gov: NCT03535298 and NCT03500328). The therapeutic options for PPMS are currently very limited with ocrelizumab being the only drug with proved efficacy and approval for this indication [8,9].

S1PR modulators are a class of oral DMTs for relapsing MS [12]. In this narrative review, we present their pharmacology, efficacy, safety profile, and risk management based on the results of phase II and phase III clinical trials. We also briefly address their impact on the risk of infections and vaccines efficacy, as well as their potential roles in treating other immune-mediated or nonimmune-mediated conditions. With this aim, we searched the English language medical literature in the PubMed database up to the 26^th^ of August 2022 using the search string ‘sphingosine 1-phosphate receptor modulators’ OR ‘S1P receptor modulators’ OR ‘S1PR modulators’ OR ‘fingolimod’ OR ‘FTY720’ OR ‘siponimod’ OR ‘BAF312’ OR ‘ozanimod’ OR ‘RPC1063’ OR ‘ponesimod’ OR ‘ACT-128800.’ Additional sources (References of PubMed articles, Google Scholar, Congresses etc.) were used for further researching specific topics (such as side effects and impact on vaccine responses).

The emphasis of our review is on the S1PR modulators already approved for the treatment of MS by North American and European regulatory agencies, but we also discuss other relevant pharmacological products. The topic is of importance, since fingolimod, the first S1PR modulator that was developed, as well as the first oral DMT approved for adults with relapsing MS, is currently the only oral DMT approved for adolescents and children with relapsing forms of MS [13–16]. Another significant breakthrough was achieved by siponimod, a second-generation, more selective S1PR modulator, that showed a modest decrease in disease progression in people with SPMS [17,18]. The latest SP1PR modulators in the MS treatment armamentarium are ponesimod and ozanimod. Considering the pleiotropism of the S1PR-related pathways, which besides peripheral immunomodulation include direct effects on CNS cells, the mechanism of action of the available S1PRs modulators in MS may be more complex than currently thought and not yet fully uncovered [17,18].

## 2. The biology of S1P signaling pathways and emerging S1PR modulators

S1P is a widely available bioactive lysophospholipid regulating an ample range of biological processes in humans and mammals [17–19]. It is a metabolite of sphingosine, one of the major constituents of myelin [20]. Erythrocytes, endothelial cells, and activated platelets are among the most important endogenous sources [21]. In the CNS, S1P is tightly regulated, being produced by sphingosine kinase and degraded by cleavage or hydrolysis [22]. Evidence suggests that its dysregulation may contribute to several diseases, including MS, atherosclerosis, and diabetes mellitus [18].

The main functions of S1P are mediated *via* five distinct transmembrane G-protein-coupled receptors (i.e. S1PR_1 –_ S1PR_5_), expressed in various amounts and combinations by most human cells [17,22–24]. Additionally, S1P may also act as secondary messenger, modulating both S1PR-dependant and S1PR-independent pathways [22].

The S1PRs activate independent intracellular pathways that are involved in lymphocyte trafficking and other immune functions (mainly S1PR_1_, but also the other S1PR subtypes) [17,23,25,26], heart rate regulation (mainly S1PR_1_ in humans, and also S1PR_3_ in mice) [25], vascular and bronchial tone [23,24], barrier permeability [23,24], microglial activation [23,24], axonal growth [20,24], neuronal plasticity and synapse formation [20,24], oligodendrocyte survival and myelination [20,27], and neurotransmitter release [22] – see Table 1 for further details [17–28]. In the extracellular space, S1P tends to accumulate in the high-density lipoprotein fraction, which possibly contributes to its anti-atherogenic effects [29].

**Table 1.** The signaling pathways of S1P and S1PR modulators.

Receptor	Main sites of expression	Biological effects of the pathway	S1PR modulators
S1PR_1_	immune cells (T cells, B cells, natural killer cells, macrophages, mast cells, dendritic cells etc.), CNS, heart, vessels, lung, lymphoid organs	lymphocyte trafficking, migration of other immune cells, degranulation of mast cells, neurogenesis, glial cells activation and proliferation, heart rate regulation, angiogenesis, endothelial barrier and blood-brain barrier permeability, smooth muscle contractions; may increase brain-derived neurotrophic factor (BDNF) in the cortex, hippocampus, and striatum of mice	Approved DMTs for MS: fingolimod, siponimod, ozanimod, ponesimod. Other compounds: ceralifimod, cerenimod, etrasimod, amiselimod, VPC01091, VPC23019a etc.
S1PR_2_	immune cells (B cells, macrophages, mast cells etc.), CNS, heart, vessels, lungs, lymphoid organs	immune cell trafficking/migration, mast cell degranulation, endothelial barrier permeability, smooth muscle contraction	Approved DMTs for MS: none Other compounds: JTE013
S1PR_3_	immune cells (B cells, macrophages, mast cells, dendritic cells etc.), CNS, heart, vessels, lungs, lymphoid organs	immune cell trafficking/migration, mast cell degranulation, endothelial barrier permeability, heart rate regulation, smooth muscle contraction	Approved DMTs for MS: fingolimod Other compounds: VPC01091, VPC23019a etc.
S1PR_4_	immune cells (T cells, B cells, macrophages, mast cells, dendritic cells etc.), CNS, lungs, lymphoid organs	immune cell trafficking/migration, bronchial smooth muscle contraction	Approved DMTs for MS: fingolimod Other compounds: amiselimod, MT1303 etc.
S1PR_5_	immune cells (natural killer cells, mast cells etc.), CNS (white matter tracts), skin, lymphoid organs	trafficking of natural killer cells, oligodendrocyte survival	Approved DMTs for MS: fingolimod, siponimod, ozanimod, ponesimod. Other compounds: cerenimod, amiselimod, KRP203 etc.

Abbreviations: CNS = central nervous system; DMT = disease-modifying treatment; MS = multiple sclerosis; S1P = sphingosine 1-phosphate; S1PR = sphingosine 1-phosphate receptor.

References: 16–38.

The available S1PR modulators are analogues of S1P that act as functional S1PR_1_ antagonists *in vivo*, initially activating the receptor but then preventing its function by inducing prolonged internalization [17,18]. The first S1PR modulator that was developed is fingolimod (FTY720), a derivative of myriocin, which is a potent inhibitor of sphingolipid synthesis with antifungal and immunosuppressant properties [17,30–32]. Notably, myriocin is a natural compound produced by the fungi *Isaria sinclairii* and *Myriococcum albomyces*, the former used in traditional Chinese medicine for promoting health and youth [30,32]. Fingolimod attracted attention because of its ability to prevent experimental graft *versus* host disease without interfering with the host *versus* leukemia response, indicating therapeutic potential for preventing graft rejection in renal transplant recipients, as well as for MS and other immune-mediated disorders, with minimal risk of infections [30,31]. The newer, second-generation, S1PR modulators that are currently approved for relapsing MS, namely siponimod (BAF312), ozanimod (RPC1063), and ponesimod (ACT-128800), were designed starting from fingolimod, aiming to improve its pharmacodynamics and pharmacokinetics to better meet therapeutic needs and mitigate the risks of side effects [17,18]. Besides MS, ozanimod has recently been approved for inducing and maintaining remission in moderate-to-severe ulcerative colitis, and clinical trials are ongoing for Crohn's disease, systemic lupus erythematosus, and Coronavirus disease 2019 (COVID-19) [17]. Concerning the latter, the S1PR_1_-mediated effects of the S1PR modulators may reduce pulmonary injury related to the excessive immune response that can be encountered in COVID-19, a potential therapeutic mechanism supported by the findings on H1N1 influenza and sepsis animal models, two conditions that also entail excessive immune activation [17,33]. Fingolimod is also assessed for several disorders other than MS, including hemorrhagic and ischemic stroke, amyotrophic lateral sclerosis, and chronic inflammatory demyelinating polyradiculoneuropathy, while siponimod is undergoing a phase II trial for intracerebral hemorrhage, and ponesimod successfully completed a phase II trial for psoriasis [17]. Additionally, fingolimod may disrupt the lifecycle of the human immunodeficiency virus (HIV), with potential therapeutic applications [34].

An impressive number of other S1PR modulators have been designed, part of them being discontinued and part currently in clinical trials for different immune-mediated disorders, such as systemic lupus erythematosus, ulcerative colitis, Crohn's disease. These include ceralifimod (ONO-4641), cerenimod (ACT-334441), etrasimod (APD334), amiselimod (MT-1303), VPC01091, and VPC23019a [17,18]. Monoclonal antibodies against S1P and inhibitors of the sphingosine kinases are also under development, preclinical data showing therapeutic potential for several cancers [17,35].

The main mechanism of action of the approved S1PR modulators for MS is preventing the infiltration of lymphocytes within the CNS by selectively depleting them from the bloodstream, without blocking their physiological activation and protective immune responses [31,36]. An overview of their characteristics and recommendations in MS can be found in Table 2. The binding of S1PR modulators results in prolonged internalization of S1PR_1_ on lymphocytes, which decreases their S1P gradient-dependant migration from the lymph nodes toward the lymphatic endothelium and then the bloodstream [17]. This process is not fully understood but is probably mediated by CCR7 retention signals. Since the egress of memory cells and effector T cells involved in the antimicrobial response is not CCR7-dependent, their exit from lymph nodes is not significantly affected by S1PR modulators, mitigating the potential risks of infections related to the use of these drugs [37]. First-dose bradycardia is related to the initial and transitory agonistic effect on S1PR_1_ and is the most common side effect of S1PR modulators. It is more pronounced in fingolimod, but is typically benign, self-limited, and avoidable for second-generation S1PR modulators by dose titration [38,39]. Other risks of S1PR modulators are also reasonably low and appear to be closely related to their receptor subtype selectivity and effects – see Table 3 and next sections for further details.

**Table 2.** Main characteristics of the S1PR modulators approved for MS.

Fingolimod* (FTY720) Novartis Pharmaceuticals, Basel, Switzerland; generic also available	Functional antagonist at S1PR_1,_ S1PR_3,_ S1PR_4,_ S1PR_5_ (significantly lower affinity for S1PR_3_).	**Trials**: FREEDOMS and FREEDOMS-II (placebo-controlled); TRANSFORMS (interferon beta-1a-controlled). **Approvals and indications**: 2010 (FDA) and 2011 (EMA) for adults with relapsing MS/highly active relapsing MS or active despite another DMT; 2018 (FDA) and 2019 (EMA) for pediatric patients 10 years and older. Decreases disease activity (clinical and brain imaging outcomes).	**Adults**: one 0.5 mg capsule, orally, once daily. **Pediatric with body weight ≤40 kg**: one 0.25 mg capsule, orally, once daily. **Pediatric >40 kg**: one 0.5 mg capsule, orally, once daily.	**Contraindications**: myocardial infarction in the previous 6 months, unstable angina pectoris, decompensated heart failure, severe heart arrhythmias requiring antiarrhythmic treatment, atrioventricular block (second-degree Mobitz II or third-degree), sick sinus syndrome without pacemaker, prolonged baseline QTc interval, stroke, or transient ischemic attack, severe lymphopenia, concurrent immunosuppression (including concomitant immunosuppressive therapies or concomitant diseases resulting in immunosuppression), ongoing cancers, severe active infections, and active chronic infections, moderate or severe liver impairment, macular edema, pregnancy or breast feeding, females with fertile potential not using effective contraceptive methods, history of PML or cryptococcal meningitis, concomitant phototherapy or photochemotherapy, untreated severe sleep apnea, hypersensitivity to the active substance or excipients. *siponimod is contraindicated in people with CYP2C9*3*3 genotypes, and reduced dose should be used in CYP2C9*2*3 or 1*3 genotypes. **Caution**: elderly patients; people with arterial hypertension, diabetes mellitus, baseline bradycardia or bradycardia-inducing concomitant medication, history of uveitis, syncope or cardiac arrest, uncontrolled hypertension, renal impairment; use of drugs that interfere with the metabolization of the S1PR modulators; concomitant use of other immunomodulators or immunosuppressant drugs; concomitant use of CYP430 inducers or other substances that may interfere with the metabolization of the S1PR modulator (e.g. St John’s Wort). **Side effects**: transient first-dose bradycardia, rarely other cardiac first-dose transient side effects (including atrioventricular block), lymphopenia (related to the mechanism of action in MS), increased liver enzymes, infections (upper respiratory and urinary tract infections, rarely VZV and HSV reactivation, and very rarely opportunistic infections, including cryptococcal meningitis and PML), hypertension, macular edema, skin cancer and very rarely other cancers, neurological side effects (convulsions, PRES), decreased efficacy of vaccines; possibly changes in ventilatory function, rebound of MS after discontinuation, paradoxical exacerbation after initiation when switching from other DMTs; birth defects and spontaneous abortions. **First dose observation (for 6 hours, or longer, if needed; heart rate, blood pressure, electrocardiogram)**: for all patients starting fingolimod; for selected patients at risk of cardiac side effects starting siponimod, ozanimod, or ponesimod. **Monitoring**: complete blood counts, liver enzymes, ophthalmology, dermatology.
**Siponimod** (BAF312) Novartis Pharmaceuticals, Basel, Switzerland	Functional antagonist at S1PR_1_, S1PR_5_	Trials: BOLD, BOLD extension, EXPAND (placebo-controlled). Approvals and indication: 2019 (FDA), for adults with relapsing MS, and 2019 (EMA) for adults with active SPMS. Decreases disease activity and disease progression (clinical and brain imaging outcomes).	Adults*: one 2 mg tables, orally, once daily; genotypes CYP2C9*2*3 or 1*3: 1 mg daily. **Titration**: over 5 days, from 0.25 mg qd to 1.25 mg qd **Pediatric**: not tested in this population	
**Ozanimod** (RPC1063) Bristol-Myers Squibb, Dublin, Ireland	Functional antagonist at S1PR_1_, S1PR_5_	Trials: RADIANCE and SUBEAM (30 mcg im qw interferon beta-1a-controlled). Approvals and indication: 2020 (FDA, EMA), for relapsing forms of MS. Decreases the annualized relapse rate and improves brain imaging outcomes compared with interferon beta-1a. Also approved for ulcerative colitis.	Adults: one 0.92 mg capsule, orally, once daily. Titration: over the course of 7 days, from 0.23 mg qd to 0.46 mg qd. **Pediatric**: not tested in this population	
**Ponesimod** (ACT-128800) Jannsen Pharmaceuticals, Beerse, Belgium	Functional antagonist at S1PR_1_, S1PR_5_	**Trials**: OPTIMUM (teriflunomide-controlled). **Approvals and indication**: 2021 (FDA, EMA), for relapsing forms of MS. Decreases the annualized relapse rate and improves brain imaging outcomes compared with teriflunomide.	Adults: one 20 mg tablet, orally, once daily. Titration: over the course of 2 weeks, from 2 to 10 mg qd. **Pediatric**: not tested in this population	

Abbreviations: DMTs = disease modifying treatments; EMA = European Medicine Agency; FDA = Food and Drug Administration; im = intramuscular; HSV = herpes simplex virus; MS = multiple sclerosis; PML = progressive multifocal leukoencephalopathy; PRES = posterior reversible encephalopathy syndrome; qw = once weekly; S1PR = sphingosine 1-phosphate receptor; SPMS = secondary progressive MS; VZV = varicella zoster virus.

References: 13–17, 40–46, 55–69.

**Table 3.** Main adverse events and mitigation strategies for the approved S1PR modulators in people with MS.

Adverse event	Suggested practical approach
Transient bradycardia	A baseline ECG should be obtained in all patients starting a S1PR modulator. First dose observation is required in patients starting fingolimod and in at-risk patients starting siponimod, ozanimod or ponesimod (i.e. patients with sinus bradycardia, or history of first- or second-degree atrioventricular block, myocardial infarction, or heart failure). All patients starting fingolimod should have the ECG and blood pressure checked prior to and 6 hours after the first dose. The heart rate and blood pressure should be checked at least hourly during the first 6 hours and whenever the patient develops new symptoms; if available, continuous ECG monitoring is recommended, otherwise, check ECG if bradycardia occurs. In patients that need medical intervention for cardiac side effects throughout the first 6 hours, overnight monitoring in a medical facility and second dose monitoring are required. If the heart rate is lowest at the end of the first 6 hours, monitoring should be extended for at least 2 hours. Same precautions are required when increasing the fingolimod dose (see Table 2, pediatric population) and when reintroducing fingolimod after a treatment interruption (depending on the duration of the interruption and the time since the start of the treatment). The monitoring required for at-risk patients starting other S1PR modulators is similar (4 hours for ponesimod, 6 hours for siponimod and ozanimod). In patients treated with beta-blockers or calcium channel-blockers that have resting heart rates below 55 bpm, discontinuation of the bradycardia-inducing drug during the initiation of the S1PR modulator, should be considered.
Atrioventricular block and other cardiac side effects	Patients with concomitant cardiac diseases that do not contraindicate S1PR modulators, should be monitored by a cardiologist during the S1PR modulator treatment and washout. Substances that may prolong the QTc interval should be avoided in patients at risk for QT prolongation (e.g. concomitant hypokalaemia).
Arterial hypertension	Blood pressure should be regularly monitored during S1PR modulator treatment and washout. If required as per current standard of care, antihypertensive medication should be introduced, or the preexisting antihypertensive regimen should be adjusted.
Macular edema	In patients at risk for macular edema (i.e. history of uveitis, diabetes mellitus), an ophthalmologic evaluation is required prior to starting the S1PR modulator and throughout the treatment. In all patients, even if asymptomatic, an ophthalmologic evaluation should be performed at 3–4 months after starting the S1PR modulator.
Liver dysfunction	Check liver enzymes and bilirubin prior to the S1PR modulator initiation. For fingolimod and ponesimod: monitor liver enzymes and bilirubin in asymptomatic patients at 1, 3, 6, 9, and 12 months after treatment initiation, and periodically thereafter. For siponimod and ozanimod: monitor liver enzymes and bilirubin periodically. Treatment should be stopped in patients with liver enzymes more than 5 times the upper limit of normal, even if asymptomatic and without signs of liver failure, and in selected patients with liver enzymes 3–5 times the upper limit of normal, depending on the results of additional investigation and presence or absence of symptoms. The S1PR modulator can be resumed after the liver enzymes return to normal if the benefits outweigh the risks, especially if an alternative case of liver injury was found.
Lymphopenia	A complete blood count should be performed before initiating a S1PR modulator and periodically thereafter (e.g. for fingolimod at 3 months and at least yearly thereafter). Peripheral lymphocyte counts may decrease to 20–30% of the baseline value. Confirmed absolute lymphocyte counts below 0.2x10^9^/l should lead to the interruption of the S1PR modulator or a reduction of dose with subsequent reassessment. The severity of lymphopenia is related to an increased risk of infections.
Infections – upper respiratory tract, urinary tract, HPV, HSV or VZV meningitis, encephalitis, or meningoencephalitis, cryptococcal meningitis, PML	A high index of suspicion for infections, including opportunistic, should be maintained during the S1PR modulator treatment and throughout the washout period. Patients should be advised to report symptoms of infections. When suspecting a serious infection, the S1PR modulator should be stopped until the infection is ruled out or cured. Specific investigations and treatments should not be delayed. After recovering from a serious infection, the possibility of restarting a S1PR modulator should be discussed with an infectious disease specialist. In the absence of a health care professional confirmed history of chickenpox or documentation of a full course of VZV vaccination, VZV antibody testing should be performed. VZV antibody negative patients are recommended to undergo a full course of vaccination before starting the S1PR modulator. A brain MRI should be performed prior to starting the S1PR modulator and periodically thereafter. Any MRI findings suggestive of PML, even in asymptomatic patients without prior natalizumab or immunosuppressant exposure, should prompt cerebrospinal fluid JCV DNA testing. Anti-JCV antibody testing in the peripheral blood prior to the S1PR modulator initiation and periodically thereafter should be considered, but negative results do not completely exclude the possibility of developing PML.
Cancers	Basal cell carcinoma and other cutaneous neoplasms, including malignant melanoma, have been reported in patients treated with S1PR modulators. Patients should be advised to be vigilant for skin lesions and to avoid sunlight exposure without photoprotection. Medical evaluation for skin lesions is recommended at S1PR treatment initiation, and every 6 to 12 months thereafter or whenever a suspicious lesion is detected; any suspicious lesion should be evaluated by a dermatologist. Cases of lymphoma and HPV-related cancers have also been reported in people treated with S1PR modulators. HPV-related cancer screening is recommended as per standard of care. If cancer is suspected or diagnosed the S1PR modulator should be discontinued.
Vaccines and decreased vaccine efficacy	Live attenuated vaccines should be avoided throughout the duration of the S1PR modulator treatment and during washout (because they may carry a risk of infection). Decreased vaccine efficacy has been observed in patients treated with S1PR modulators, especially fingolimod. Vaccination against VZV in antibody negative patients, and other vaccinations as per standard of care, including HPV, are recommended before starting the S1PR modulator treatment (see above). The benefits and risks of discontinuing fingolimod or other S1PR modulators to allow for effective vaccination should be considered on a case-by-case basis (see below).
Spontaneous abortion and birth defects	Female patients of childbearing potential should be informed of the teratogenic risks of S1PR modulators, should have a negative pregnancy test when starting the S1PR modulator, and should use effective contraception methods that should be maintained during the washout period after treatment discontinuation. The outcome of any pregnancy in patients using S1PR modulators should be reported.
Paradoxical MS exacerbation (tumefactive lesions)	Rare cases of paradoxical MS exacerbation with tumefactive lesions associated with MS relapse were reported at S1PR initiation (e.g. when switching from beta-interferons to fingolimod). In case of severe relapses occurring after S1PR initiation, a brain ± spinal cord MRI should be obtained. If a tumefactive lesion is found, discontinuation of the S1PR modulator should be considered on a case-by-case basis.
Other unexpected neurological manifestations	In the presence of neurological (or psychiatric) symptoms and signs that are not compatible with a typical MS exacerbation, a brain ± spinal cord MRI should be obtained as soon as possible; CSF examination and other investigation may also be warranted. Besides the above mentioned neuroinfections and tumefactive MS lesions, cases of PRES have been reported in patients using S1PR modulaters. In case of PRES, the S1PR modulator should be discontinued.
Return of disease activity or MS rebound	MS rebound after the discontinuation of S1PR modulator treatments has been reported. Patients on S1PR modulators should be advised against ‘drug holidays.’ Caution should be taken when recommending transient treatment discontinuation – e.g. for vaccination purposes.

Abbreviations: bpm = beats per minute; CSF = cerebrospinal fluid; DMTs = disease modifying treatments; DNA = deoxyribonucleic acid; ECG = electrocardiogram; HPV = human papilloma virus; HSV = herpes simplex virus; JCV = John Cunningham virus; MRI = magnetic resonance imaging; MS = multiple sclerosis; PML = progressive multifocal leukoencephalopathy; PRES = posterior reversible encephalopathy syndrome; S1PR = sphingosine 1-phosphate receptor; VZV = varicella zoster virus.

References: https://www.ema.europa.eu/en/documents/product-information/gilenya-epar-product-information_en.pdf (25.9.22); https://www.ema.europa.eu/en/documents/product-information/mayzent-epar-product-information_en.pdf (25.9.22); https://www.ema.europa.eu/en/documents/product-information/ponvory-epar-product-information_en.pdf (25.9.22); https://www.ema.europa.eu/en/documents/product-information/zeposia-epar-product-information_en.pdf (25.9.22).

Interestingly, the brain expresses very high levels of S1PR_s_, both on the membranes of neurons, and on glial cells [22]. The approved S1PR modulators easily pass the blood-brain barrier (BBB), so it is biologically plausible that they could promote neuroprotection, neurorepair, neuroregeneration, and remyelination by binding receptors on astrocytes and oligodendrocytes, as well as have local immunomodulatory effects by binding receptors on the microglia [17,36]. In this respect, experimental data show that different S1PR modulators may have distinct effects on the CNS neurons and glial cells depending on their S1PR_1_ and S1PR_5_ selectivity and downstream transcriptional effects [18]. For example, fingolimod may promote neuroprotection, neurorepair, and neuroregeneration [30,40], and siponimod may limit demyelination, possibly by prolonging the survival of mature oligodendrocytes by its effects on S1PR_5_ [27,41]. Preclinical data also suggest possible beneficial effects S1PR modulators in Parkinson’s disease and Alzheimer’s disease, as well as in other neurodegenerative disorders [17]. Concurrently, S1PR modulators could also regulate the BBB permeability, providing additional therapeutic benefits in MS [17,27]. In terms of clinical efficacy, siponimod proved a mild effect in decreasing disability progression in people with active SPMS, which appears independent of its effect on relapses, possibly suggesting a clinically relevant neuroprotective effect [42].

The approved S1PR modulators and their efficacy in MS are further discussed in the next sections – also see Table 2. The impact of the S1PR modulators used in MS on the risk of infections and vaccines efficacy – topical in the context of COVID-19, as well as the other safety concerns and mitigation strategies which are mostly class-related, are also discussed for all the approved S1PR modulators separately.

## 3. Fingolimod

Fingolimod, also known as FTY720 during its development and clinical trials, is the first S1PR modulator that was discovered, and the first oral drug approved as a DMT for MS [30]. It is currently approved in the USA for adults, adolescents, and children over 10 years of age with relapsing MS, and in the EU and UK for adults, adolescents, and children with highly active RRMS or with RRMS that remains active despite treatment with another appropriate DMT [14–18,43] – see Table 2. It is also approved for similar indications in many other countries and regions worldwide. The approvals are based on the results of FREEDOMS, FREEDOMS-II, and TRANSFORMS, the pivotal trials of fingolimod in adults with RRMS, showing that fingolimod significantly reduces relapses and delays disability accrual and brain atrophy compared to placebo and intramuscular interferon beta-1a, respectively [14,44,45], and on the results of the PARADIGMS trial for pediatric relapsing MS [16]. INFORMS, a large phase III clinical trial, failed to prove the efficacy of fingolimod in PPMS in terms of disability progression and brain volume loss compared to placebo [46]. However, a *post hoc* analysis of the INFORMS subgroup that had inflammatory activity at baseline found a delay in brain volume loss with fingolimod, suggesting that one of the reasons the trial failed could be that most of the trial population was outside the therapeutic window [47]. Fingolimod is a prodrug, requiring phosphorylation by cellular sphingosine kinases in order to gain affinity for S1PRs [48]. Phosphorylated fingolimod acts as a functional S1PR_1_ and S1PR_3 –_ S1PR_5_ antagonist, internalizing the S1PR_1_ on lymphocytes, sequestering them within the lymph nodes, and preventing their infiltration within the CNS, the main mechanism of action in MS [30,37].

The effects of fingolimod on S1PR_1_ also explain its most common side effects: transitory bradycardia, atrioventricular block, arterial hypertension, and macular edema, the last two also being mediated by S1PR_3_ in humans [17]. On the other hand, the egress from lymph nodes of memory and effector cells involved in antimicrobial responses appears to be unaffected by the S1PR_1_ antagonism, therefore this mechanism does not explain the increased risk of infections and potential reduction in vaccine efficacy observed with fingolimod [49–55].

Besides the S1PR_1_-dependent impairment of lymphocyte trafficking, fingolimod also interferes with S1PR_5_ on astrocytes and other CNS cells, experimental studies suggesting potentially beneficial neuroprotective effects, though their clinical relevance is debatable [30,40]. However, the expression of sphingosine kinases in the cortex of people with progressive MS could be lower than in people with relapsing-remitting disease, which could in turn result in lower CNS levels of phosphorylated fingolimod, suggesting a potential advantage of the newer, second-generation S1PR modulators in progressive MS [47].

Compared to the second-generation S1PR modulators, fingolimod has a longer elimination half-life, probably related to its phosphorylation [21,49]. This makes fingolimod, theoretically at least, a useful drug for patients who occasionally ‘miss their tablet.’ Interestingly, unphosphorylated fingolimod also seems to have biological effects independent of the S1PR_s_ pathways, blocking the arachidonic acid phospholipase A2 signaling, with inhibitory consequences on cytotoxic T cells [37]. While this is a potential complementary mechanism of action in MS, it could also explain the higher risk of viral infections and the potentially reduced vaccine efficacy observed with fingolimod [37,49–55] (see section 7). Fingolimod is slowly eliminated, mainly by CYP3A4 hepatic metabolization, with a washout period of about 2 months. CYP3A4 inducers, such as carbamazepine and modafinil, which are sometimes used as symptomatic treatment in MS, can slightly increase the elimination of fingolimod, therefore decreasing drug exposure; while potent CYP3A4 inhibitors, such as ketoconazole, block its hepatic metabolization, increasing the exposure to fingolimod and potentially the risk of side effects [17,18]. The impact of these drug interactions in clinical practice appears to be minimal, but precaution is warranted [18].

The contraindications for fingolimod overlap with those of the other approved S1PR modulators and include myocardial infarction in the previous 6 months, unstable angina pectoris, heart failure, heart arrhythmias requiring antiarrhythmic treatment, first and second degree atrioventricular block, sick sinus syndrome without pacemaker, prolonged baseline QTc interval, stroke and transient ischemic attack, ongoing cancers, severe active infections and active chronic infections, liver impairment, and macular edema [8–10,12–16]. Considering the increased risk of spontaneous abortion and malformations, fingolimod is contraindicated during pregnancy and in female patients of fertile age that do not use effective contraception methods [8–10]. Fingolimod can cross the placenta, and may have teratogenic effect in rats (persistent truncus arteriosus and ventricular septal defects) [56]. In the clinical development fingolimod program [57] which included 89 exposed pregnancies (i.e. fingolimod ongoing at conception or 6 weeks before), spontaneous abortion occurred in 24% of pregnancies and abnormal fetal development in 7.6% of cases (slightly higher, and borderline normal, respectively, when compared to the rate registered in the general population) [57]. In another 717 pregnancies exposed to fingolimod, the prevalence of major cardiac abnormalities and spontaneous and elective abortion was comparable with that in the general population [58].

The most common adverse events observed with fingolimod include first-dose bradycardia (requiring first-dose observation), lymphopenia (related to the mechanism of action in MS), and infections, usually upper respiratory or urinary tract [14–18]. Cases of varicella-zoster virus (VZV) and herpes simplex virus (HSV) reactivation resulting in meningitis, encephalitis, or meningoencephalitis, as well as cases of fungal meningitis, including cryptococcal, have been reported. A review of cases of cryptococcal meningitis in the Novartis safety database identified until February 2020, 60 case reports (estimated rate 8/100,000 patient-years), of which 13 had a fatal outcome [59]. Of particular interest is progressive multifocal leukoencephalopathy (PML), a demyelinating opportunistic infection caused by the reactivation of the John Cunningham (JC) virus, cases being reported in patients treated with fingolimod even without prior natalizumab exposure [14–17]. A systematic review and meta-analysis of PML cases in people with MS treated with DMT covering the cases reported until December 2020, identified fingolimod as ranking second after natalizumab, with 20 cases of PML reported [60]. Interestingly, fingolimod-associated PML patients showed less disability progression related to the disease as measured by worsening of the EDSS score ≥1.0, compared with the other patients with PML on other DMTs [60]. Increased liver enzymes, with a few cases of liver failure, arterial hypertension, macular edema, skin or other cancers, and decreased efficacy of vaccine have also been reported [17,18]. Practical recommendations for risk mitigation in clinical settings are discussed in section 8 and Tables 2 and 3.

## 4. Siponimod

Siponimod, also known as BAF312, is a second-generation S1PR modulator, highly selective for S1PR_1_ and S1PR_5_. *In vivo* it acts as a potent functional S1PR_1_ antagonist, inducing prolonged internalization of S1PR_1_ [21,36]. It is the second S1PR modulator to enter clinical trials for MS and the first DMT to prove efficacy, albeit modest, in decreasing confirmed disability progression, and the time until transitioning to the use of a wheelchair and brain volume loss in people with SPMS, reducing the annualized relapse rate and magnetic resonance imaging signs of disease activity [42]. The main mechanism for siponimod’s effects appears to be reducing brain inflammation. In a post-hoc analysis of the phase III EXPAND trial, the effect on confirmed disability progression was more pronounced in active SPMS than in all patients in the original phase III trial [61]. However, siponimod has also an effect on MRI measures relevant to neurodegeneration (i.e. gray matter atrophy, magnetization transfer ratio relevant to myelin density) both in people with active or non-active SPMS [62]. Therefore, siponimod shows a greater effect on clinical outcomes in the active SPMS group, but a similar response in the overall or non-active SPMS groups, on these MRI measures which may pertain to neurodegeneration and tissue integrity [62], thus suggesting that it may work through two possibly interconnected pathophysiological mechanistic pathways affecting inflammation and neurodegeneration [61]. Early treatment initiation is associated with long-term benefit [63]. It may also have a positive impact on cognition [61,64]. In the USA, siponimod is approved for adults with relapsing MS, ranging from CIS to SPMS, while in the EU is only approved for SPMS. The approvals are based on the placebo-controlled phase II BOLD trial and its extension, for RRMS [65,66], and the phase III placebo-controlled EXPAND trial, for SPMS [42].

Structurally, siponimod is an alkoxyimino derivative of fingolimod, developed by optimizing the initial structure to increase its potency for S1PR_1_ and selectivity against S1PR_3_, thus increasing the therapeutic potential for MS and decreasing the risks of side effects [21]. Pharmacokinetics were also improved, allowing for administration in a single daily dose and rapid restoration of lymphocyte counts in the peripheral blood after discontinuation [21].

As in the case of the other S1PR modulators, the main mechanism of action of siponimod in MS, as well as the most common side effects, are related to its effects on S1PR_1_ [42] (see section 8 and Tables 2 and 3 for side effects and practical recommendations for risk mitigation). Compared to fingolimod, the chronotropic first-dose effects of siponimod are milder and avoidable by drug titration, most patients not requiring first dose observation [42]. For at risk patients (i.e. patients with sinus bradycardia, or history of first- or second-degree atrioventricular block, myocardial infarction, or heart failure), first-dose observation is required, similarly as in the case of fingolimod (see Table 3).

Siponimod easily passes the BBB and has potential neuroprotective effects, mediated via S1PR_5_ [12]. Serum neurofilament-light chains levels in patients with active and non-active SPMS receiving siponimod are lower than in those with placebo, suggesting lower neuronal damage [18]. The average time for peripheral blood lymphocyte counts decline is 4–6 hours after the first dose and restoration to lower reference range levels takes up to 10 days after its discontinuation [21,36]. Siponimod is metabolized mainly by CYP2C9, and to a much lesser degree by CYP3A4. Genetic polymorphism associated to reduced CYP2C9 enzymatic activity results in higher systemic siponimod exposure, and in people with CYP2C9*3*3 siponimod is contraindicated [67,68] Despite the small role of CYP3A4, drugs that induce CYP3A4, such as carbamazepine, may decrease siponimod exposure, while drugs that inhibit CYP3A4 (e.g. ketoconazole) increase the exposure to siponimod, their concomitant use requiring additional precautions [18].

Siponimod and its metabolites can cross the placenta, and can have toxic effects on the embryo and fetus in rats and rabbits, and induced teratogenicity in rats [69]. The data on the use of siponimod in pregnant patients is limited. Based on the animal studies, treatment with siponimod is contraindicated during gestation and in fertile women who do not use effective contraception [70].

## 5. Ozanimod

Ozanimod (RPC1063) is a second-generation selective S1PR_1_ and S1PR_5_ agonists that acts as functional S1PR_1_ antagonist *in vivo*. It is currently approved for relapsing MS in the USA and the EU, and partially available in the UK (i.e. not available in England and Wales; available in Scotland; the decision for Northern Ireland is pending) [71]. The approval is based on the results of RADIANCE, a phase II/III placebo and interferon beta-1a controlled trial [72,73], and SUNBEAM, a phase III interferon beta-1a controlled trial showing a good safety profile, reduction of the annualized relapse rate, and improved brain imagistic outcomes compared with interferon beta-1a [74,75]. The effects of ozanimod on delaying disease progression are biologically plausible and supported by a reduction in the brain volume loss, but the SUNBMEM trial did not prove efficacy in delaying confirmed disability progression, a topic that requires further study [74–76].

The mechanism of action and safety concerns of ozanimod are shared with that of other S1PR modulators [72–76] (see section 8 and Tables 2 and 3). The metabolism of ozanimod is complex, with a shorter elimination half-life compared to siponimod [18,76]. Despite being pharmacologically active, the administration of ozanimod results in several active metabolites that require CYP3A4 for activation and inactivation and MAO-B for activation [18]. Drugs that induce CYP3A4 appear to increase the metabolization of ozanimod (therefore decreasing drug exposure), and CYP3A4 inhibitors increase ozanimod exposure. MAO-B inhibitors may reduce the exposure to active metabolites, reducing the clinical efficacy of the drug. The concurrent use of drugs that induce or inhibit CYP3A4, as well as of MAO inhibitors, should be avoided [18].

## 6. Ponesimod

Ponesimod (ACT-128800) is a highly selective S1PR_1_ functional antagonist with some S1PR_5_ activity. It is currently approved for relapsing MS in the USA and the EU, and available in the UK [76,77]. Its approval was based on the results of a placebo-controlled phase IIb trial [39], and an active-controlled phase III trial (OPTIMUM) [78], demonstrating a good safety profile and superiority over teriflunomide, the second oral DMT approved for MS [79], in reducing the annualized relapse rate, fatigue, cerebral magnetic resonance imaging activity, and brain volume loss, and in achieving and maintaining no evidence of disease activity [39,78]. Like ozanimod, ponesimod did not prove efficacy in reducing confirmed disability progression, but reduced brain volume loss [78]. The mechanism of action and safety concerns are shared with the other S1PR modulators, and dose titration mandates the need for first-dose cardiac monitoring [39,78]. The reversibility of the peripheral blood lymphocyte counts takes up to a week, being lower than with the other S1PR modulators [39,78,80]. Ponesimod undergoes limited metabolization. Ponesimod and its metabolites are unlikely to show any clinically relevant drug-drug interaction potential for CYP or UGT enzymes, or transporters [18]. Side effects and risk mitigation are mentioned in section 8 and Tables 2 and 3.

## 7. Infections and vaccination in people with MS treated with S1PR modulators

The S1PR_1_ modulators approved for MS induce a more pronounced depletion of naïve and helper T cells in the peripheral blood compared to cytotoxic T cells, central memory T cells and peripheral effectors memory T cells, which should generally confer a favorable safety profile in respect to infections [17,18,36,37]. However, their use (and especially in the case of fingolimod) can be associated to an increased risk of infections, mainly upper respiratory and urinary tract [17,18]. Cases of PML have been reported in people with MS treated with fingolimod and other S1PR modulators, even in the absence of prior natalizumab exposure, a DMT known for its higher risk of PML, with an age-related incidence, which may impact consideration of use in certain populations [17,18,81]. Nevertheless, the estimated risk and incidence rate remain very low in people with MS without prior immunosuppressant use or previous exposure to natalizumab [81]. The risks of VZV and HSV reactivation/infection, cryptococcal meningitis, and other opportunistic fungal or bacterial CNS infections, seem very low in people with MS treated with S1PR modulators, with similar or slightly higher incidence rates and outcomes compared to placebo [82]. Particular caution is needed in populations with higher risk (e.g. elderly patients, concomitant diabetes mellitus) and with longer duration of treatment [59]. A case of disseminated VZV infection was reported with fingolimod, leading to the recommendation to check VZV antibodies and, if needed, vaccination, before the initiation of treatment with S1PR modulators [45].

Given their selectivity in lymphocyte depletion, the impact of S1PR modulators on vaccine efficacy is expected to be lower than with some other DMTs. A randomized study on the efficacy of influenza and pneumococcal polysaccharide vaccination, models for T-cell-dependent and -independent vaccinations, respectively, in people taking siponimod, found that seroprotection for influenza was achieved in about 70%, while seroprotection for Pneumococcus was achieved in more than 90% [83].

The ongoing Severe Acute Respiratory Syndrome Coronavirus 2 (SARS-CoV-2) pandemic raised safety concerns in people with MS treated with DMTs regarding their risk of getting infected, their risk of severe COVID-19, and the efficacy of the recently developed SARS-CoV-2 vaccines [84]. The available data suggest that people with MS on fingolimod treatment who were infected by SARS-CoV-2 had mostly asymptomatic or mild COVID-19, and those with severe COVID-19 recovered more rapidly than expected [85], though the recent data following the vaccination campaign suggest worse outcomes [86]. A recent study in England found that the risk of SARS-CoV-2 infection increased significantly following the relaxation of COVID-19 restrictions in people with MS treated with fingolimod or anti-CD20 monoclonal antibodies, compared with the general population, despite mass vaccination, reflecting decreased vaccine efficacy (see further data, on subgroups of the same population, below), and possible higher risks of infections with these DMTs [55]. The same study found that beta-interferons, another class of DMTs used in MS, were associated with a lower risk of SARS-CoV-2 infection than that of the general population [55], not surprisingly, given their antiviral properties [87]. After the viral replication phase comes to an end, the severity of COVID-19 is driven by a hyperinflammatory response, which is part of the rationale of studying the effects of ozanimod in COVID-19 [17,33]. Nevertheless, potential risks posed even by short-acting S1PR modulators when used as immunomodulators in the context of an ongoing SARS-CoV-2 infection may be greater than those posed by beta-interferons, and further studies are needed [55,87].

Fingolimod seems to prevent the production of antibodies in response to SARS-CoV-2 vaccination, decreased seroconversion rates being observed in people with MS treated with fingolimod or anti-CD20 monoclonal antibodies, compared to those without DMT [51,53,54,88]. A detectable anti-SARS-CoV-2 peripheral T cell response, which arguably could confer a degree of protection, was found in less than a half of those with absent humoral response, and detectable peripheral T cell-responder rates were much lower in the fingolimod group than in the anti-CD20 group (1 out of 6 patients versus 4 out of 8) [51]. A subsequent study on the same population found that the vaccine booster dose resulted in seroconversion in most people treated with fingolimod that were previously seronegative, but fails to elicit an anti-SARS-CoV-2 T cell response [52].

Since the other S1PR modulators are not yet widely used in clinical practice (compared to fingolimod), real-world evidence is very limited or absent for siponimod, ponesimod, and ozanimod. Despite being plausible to consider the above observations, a class effect and extrapolate the results on fingolimod, as mentioned above, fingolimod may result in higher cytotoxic T cell impairment compared with other S1PR modulators [37], which could contribute to its impact on vaccines efficacy.

Temporary withdrawal of the S1PR modulator treatment was suggested as a strategy to facilitate successful vaccination, moreover in the case of ponesimod and siponimod, which have shorter times until peripheral blood lymphocyte restoration [39,78,80]. However, considering the risk of MS rebound/reactivation reported in a few cases of S1PR modulator cessation, the safety and risk/benefit ratio of such an approach warrants further study [51,74,75,80], and this should include identifying people with MS at risk for rebound after stopping fingolimod. In a study on 992 RRMS patients treated with fingolimod for 6 months or more [89], 12.5% of the patients had clinical rebound and only a minority of those (3.6%) had clinical rebound without disease activity before discontinuation [89]. In this study, disease activity before discontinuation, female sex, and younger age were associated with a higher relapse risk after discontinuation [89].

## 8. Considerations on other risks and risk mitigation strategies in people with MS treated with S1PR modulators

S1PR modulators induce dose-dependent bradycardia, reaching a maximum in the first hours after administration and declining significantly and disappearing at subsequent doses [36,90]. Despite being a class effect, bradycardia is more pronounced with fingolimod, which requires first-dose observation, with hearth rate and electrocardiogram monitoring. In second-generation S1PR modulators, the bradycardia is mitigated by dose titration, so first-dose observation is not required, except in selected cases when starting siponimod [72,74,90]. Other first-dose cardiac side effects, albeit rare, include transient first and second degree atrio-ventricular bloc [36,42,65,90]. Same precautions as those used at initiation should be taken when resuming the S1PR modulator after a longer pause, or when increasing the fingolimod dose to adjust it to the body weight in pediatric patients [72,74,90].

Several cardiac diseases contraindicate the use of S1PR modulators, so cardiologic evaluation for those with cardiac disease history is advised prior any S1PR modulator initiation. S1PR modulators cause vasoconstriction and decrease endothelial-derived nitric oxide levels, which may result in arterial hypertension [36,90–92]. Macular edema has also been observed with the use of S1PR modulators and is probably related to their effect on vascular permeability [36,90,91]. Although clinically significant changes are not common, periodical cardiologic and ophthalmologic monitoring is required, more tightly in people with hypertension or diabetes, and other vascular risk factors [91].

S1PR1 is also involved in the recruitment of CD8+ cytotoxic T cells and natural killer cells into tumors. The potential impact of the long-term use of S1PR modulators on the risk of cancer and on cancer prognostic appears to be low, but needs further clarification [91]. Available data, however, suggest an increased risk of skin cancer in S1PR modulators users, especially basal cell carcinoma, and possibly also melanoma [93]. Considering this, dermatologic evaluation is needed prior to treatment initiation and periodically thereafter [17,18,93].

Siponimod is extensively metabolized by CYP2C9, genetic polymorphism associated to reduced CYP2C9 enzymatic activity resulting in significantly higher siponimod exposure [67,68]. Thus, genetic testing is required prior to siponimod initiation, the presence of CYP2C9*3*3 being a contraindication for siponimod, while the CYP2C9*3*1 and CYP2C9*3*2 genotypes require reduce dosage [17,18]. Metabolization of the other approved S1PR modulators is not dependent upon a single cytochrome system, so pharmacogenetic testing is not necessary. The concomitant use of drugs that can interfere with the metabolization of the S1PR modulators requires caution [17,18]. Further details on contraindications, side effects, and risk mitigation strategies can be found in Table 2.

A case of paradoxical MS exacerbation occurring after the initiation of siponimod was reported in a patient with SPMS previously treated with dimethyl fumarate [94]. The mechanism is not clear, a potential explanation being a rapid change in the helper versus cytotoxic T cell ratio or possibly the expansion of the cytotoxic T cells with myelin basic protein reactive phenotypes [94–96]. Stopping fingolimod has rarely been associated with severe MS rebound, including a few fatal cases, with unclear mechanism, possibly related to massive release of lymphocytes from lymph nodes and concomitant overexpression of S1PR_s_ on astrocytes and other CNS cells [97]. Considering this, precaution should be taken when switching the S1PR modulators with or from another DMT, or when stopping them without initiating another DMT.

## 9. Conclusion

The S1PR modulators are an evolving class of DMTs for relapsing MS, with good efficacy and safety profiles provided precaution measures are followed. Four drugs are currently approved for adults with relapsing MS, fingolimod, the first-in-class, siponimod, ozanimod, and ponesimod, fingolimod also being approved for children and adolescents. Their main mechanism of action in MS is blocking the S1P-dependent egress of lymphocytes from lymph nodes into the bloodstream by binding and internalizing the S1PR_1_ on lymphocytes, thus limiting lymphocyte infiltration in the CNS. The more selective, second-generation S1PR modulators, maintain good efficacy in decreasing disease activity, have a more favorable safety profile, and shorter washout periods. All S1PR modulators easily cross the BBB, and may have neuroprotective, neuroregenerative, and promyelinating effects related to their direct CNS activity mediated *via* S1PR_5_. Further research is needed to better harness their full therapeutic potential and refine their use in MS.

## 10. Expert opinion

S1PR modulators pose several advantages among the available DMTs for MS: a convenient route of administration, good tolerability, a good safety profile provided precaution measures are followed, and a theoretical potential to delay the neurodegenerative component of MS, independent of decreasing the activity of the disease. However, no comparative data between the different S1PR agents exists in this regard, all of which are similarly classified with respect to indication by the US FDA. Given its modest efficacy data in SPMS in patients without inflammatory disease markers, the superiority of siponimod (and by extension ozanimod – which affects the same receptor subtypes) over fingolimod in limiting neurodegeneration is hardly established, while for ponesimod, which predominately affects S1PR_1_, the rationale for a CNS effect is debatable.

The oral dosing is an advantage, as shown by the improved patient self-reported outcomes in people with MS who switched directly from an injectable DMT to fingolimod without a washout [98]. At the same time, fingolimod is one of the few options available for the pediatric population with MS. S1PR modulators also have therapeutic potential for other immune- and nonimmune-mediated disorders, ozanimod already proving efficacy in ulcerative colitis, which may sometimes coexist with MS.

In respect to within-class comparisons, no head-to-head trials were performed. Since the designs of the pivotal trials and the populations enrolled were quite different, it would be hazardous to argue the superiority of one drug over another in reducing MS activity. On the other hand, siponimod is the only one that proved efficacy in delaying confirmed disability progression in people with SPMS.

Although traditionally not routinely assessed in the MS clinic, affected cognition is a frequent consequence of MS. Interestingly, siponimod has a significant benefit on cognitive speed processing measured by the single-digit-modality-test (SDMT) in people with SPMS [99]. While the cited study has its own limitation (as identified by Leavitt and Rocca [64], i.e. cognitive measures were not listed amongst the initial secondary outcomes of the study; choice of the type of testing; the interpretation of the lesser decline in SDMT performance in those study participants with more advanced disease), it does incite to further mechanistic considerations on siponimod actions at the level of the BBB [100] or beyond [101].

Fatigue is a symptom commonly encountered in people with MS, but sometimes neglected in clinical trials. Ponesimod proved superiority in reducing fatigue compared to teriflunomide [39,78], as measured with the Fatigue Symptom and Impact Questionnaire-Relapsing Multiple Sclerosis (FSIQ-RMS). Currently, it is not clear if this is a class effect, neither if the reduction in fatigue in people treated with ponesimod in the trial was in fact associated to ponesimod’s effect on reducing CNS inflammation (a reduction of combined active MRI lesions i.e. new Gd+ T1 plus new or enlarging T2 lesions, by 56% compared with teriflunomide).

Fingolimod is the first-in-class and the least selective S1PR modulator in respect to its S1PR subtype affinity, which explains at least in part its somewhat more challenging safety profile. It is also a prodrug, requiring phosphorylation to become a S1PR modulator. Interestingly, this may confer it additional mechanisms of actions that could add to its therapeutic benefits in MS in terms of disease activity, the unphosphorylated form interfering with the arachidonic acid pathway, and decreasing the activity of cytotoxic T cells. This could also explain its apparently higher risk of viral infections and negative impact on vaccines efficacy. Moreover, the need for phosphorylation in other to act on the CNS S1PR_5_, which might be decreased within the CNS of people with more advanced disease, could explain its failure to prove efficacy in delaying disability progression in people with PPMS. More real-life data is needed to clarify if and what are the factors which are associated with rebound post-fingolimod, which were suggested by some [102] but not confirmed by others [103]. Siponimod is the only S1PR modulator to prove clinically significant efficacy in people with active SPMS, by reducing confirmed disability progression unrelated to relapses or magnetic resonance imaging activity, as well as the time until the need to use a wheelchair. The only other drug that proved efficacy in delaying disease progression unrelated to relapses in clinical studies, is ocrelizumab, an anti-CD20 monoclonal antibody, which poses different safety concerns than siponimod. Owing to its dependence on a single cytochrome system instead of multiple pathways, siponimod requires genetic testing prior to initiation, which is easily accessible in the clinical practice settings in which siponimod is available. The potential long-term benefits of using siponimod in delaying or preventing the secondary progressive phase of MS are plausible but remain to be proven.

While both fingolimod and ozanimod proved superiority over interferon beta-1a in clinical trials, ponesimod is currently the only S1PR modulator to being compared with another oral DMT (proving superiority to teriflunomide). This, along with its short washout, could make ponesimod the preferred S1PR modulator for people switching from teriflunomide, and also for other people with relapsing forms of MS.

Preclinical data indicate that all the approved S1PR modulators have potential for neuroprotective, neuroregenerative, and pro-myelinating effects mediated by S1PR_5_, but these may not be identical between compounds. All of them proved reduction in brain volume loss, but except for siponimod as already mentioned, failed to prove efficacy in delaying confirmed disability progression unrelated to relapses. Further clinical data, also from post-marketing studies, are needed to inform this issue, as well as any personalized medicine aspects (are some specific individuals to respond best to a specific S1PR modulator?). Based on the current data and allowing the current regional approvals which set criteria for eligibility for treatment, siponimod seems the more rational choice for people transitioning toward the secondary progressive phase and for people with SPMS, while fingolimod may pose certain advantages in people with highly active relapsing MS, provided they are at low risk for side effects. Again, subject to their availability and label registration, ozanimod and ponesimod are good choices for people with relapsing MS with genotypes that preclude the use of siponimod, and good alternatives to fingolimod in view of their safety profile (especially in people with concomitant conditions that increase the risks of using fingolimod).

The current COVID-19 pandemic, and its probable aftermaths (cohabitation with SARS-CoV-2, need of possible periodic vaccinations), sheds a new light on the immunological effects of the S1PR modulators. A recent meta-analysis shows that response to vaccination is blunted in 70% of people with MS treated with S1PR modulators [88]. These data mainly refer to fingolimod, and careful prospective studies are needed to assess if this is a class effect. Recent data suggest the risk of more severe COVID-19 in patients receiving fingolimod or siponimod seems to be similar to that reported in the general population and the MS population with COVID-19 [104]. Whilst the frequency of COVID-19 is higher in patients with MS on fingolimod, extrapolating this to the newer S1PR is intellectually tempting but needs further careful clinical observation, not least because the severity of COVID-19 itself has changed, and different viral strains may be responsible of different clinical COVID-19 phenotypes. A careful post-marketing surveillance and audit effort is therefore needed, not only with the traditional focus on MS disease response to S1PR modulators but also on their immunological effects. Hence, a ‘silver lining’ consequence of the post-pandemic is neurologists taking heed of immunological effects of the MS drugs they currently use and thus understanding them better, to the benefit of people with MS. S1PR modulators seem to represent such a case-study.

## Article highlights

- Sphingosine 1-phosphate (S1P) receptor modulators are a class of oral disease-modifying therapies (DMTs) for relapsing forms of multiple sclerosis (MS).
- S1P is an endogenous bioactive lipid. The S1P signaling pathways regulate lymphocyte trafficking, vascular permeability, vascular and bronchial smooth muscle tone, and various neurologic and cardiac functions.
- The currently approved S1PR modulators for relapsing MS are fingolimod (the first-in-class), siponimod, ozanimod, and ponesimod. Ozanimod is also approved for the treatment of ulcerative colitis.
- Fingolimod is the first oral DMT approved for adults with MS, and the first and only oral DMT approved for pediatric patients with MS, aged 10 years and older.
- Siponimod is the first and only drug with proved modest efficacy in delaying confirmed disability progression, independent of disease activity, in people with active SPMS. In the European Union, siponimod is only approved with this indication.
- Ponesimod is the first oral DMT for MS that proved efficacy against another oral DMT for MS, namely teriflunomide.
- The approved S1PR modulators are generally safe, but first-dose observation for bradycardia and atrioventricular block is required when starting fingolimod, while dose titration is typically enough for siponimod, ozanimod and ponesimod. All these drugs require careful patient selection, monitoring for potentially serious adverse events, and precautions for drug interactions. The potential negative impact on the response to certain vaccines and the risk of infection should be considered.

This box summarizes key points contained in the article.

## References

[1] Lublin FD, Reingold SC, Cohen JA, et al. Defining the clinical course of multiple sclerosis: the 2013 revisions. Neurology. 2014 Jul 15;83(3):278–286. 24871874 10.1212/WNL.0000000000000560 PMC4117366

[2] Walton C, King R, Rechtman L, et al. Rising prevalence of multiple sclerosis worldwide: insights from the Atlas of MS, third edition. Mult Scler. 2020 Dec;26(14):1816–1821. 33174475 10.1177/1352458520970841 PMC7720355

[3] MS T. How common is MS? [cited 2022 Aug 1]. Available from: https://mstrust.org.uk/a-z/how-common-multiple-sclerosis

[4] Collaborators GBDMS, Culpepper WJ, Nichols E. Global, regional, and national burden of multiple sclerosis 1990-2016: a systematic analysis for the global burden of disease study 2016. Lancet Neurol. 2019 Mar;18(3):269–285. 30679040 10.1016/S1474-4422(18)30443-5 PMC6372756

[5] Luchetti S, Fransen NL, van Eden CG, et al. Progressive multiple sclerosis patients show substantial lesion activity that correlates with clinical disease severity and sex: a retrospective autopsy cohort analysis. Acta Neuropathol. 2018 Apr;135(4):511–528. 29441412 10.1007/s00401-018-1818-y PMC5978927

[6] Lublin FD, Reingold SC. Defining the clinical course of multiple sclerosis: results of an international survey. National multiple sclerosis society (USA) advisory committee on clinical trials of new agents in multiple sclerosis. Neurology. 1996 Apr;46(4):907–911. 8780061 10.1212/wnl.46.4.907

[7] Thompson AJ, Banwell BL, Barkhof F, et al. Diagnosis of multiple sclerosis: 2017 revisions of the McDonald criteria. Lancet Neurol. 2018 Feb;17(2):162–173. 29275977 10.1016/S1474-4422(17)30470-2

[8] Montalban X, Gold R, Thompson AJ, et al. ECTRIMS/EAN guideline on the pharmacological treatment of people with multiple sclerosis. Eur J Neurol. 2018 Feb;25(2):215–237. 29352526 10.1111/ene.13536

[9] Rae-Grant A, GS D, RA M, et al. Practice guideline recommendations summary: disease-modifying therapies for adults with multiple sclerosis: report of the guideline development, dissemination, and implementation subcommittee of the american academy of neurology. Neurology. 2018 Apr 24;90(17):777–788. 29686116 10.1212/WNL.0000000000005347

[10] Scolding N, Barnes D, Cader S, et al. Association of british neurologists: revised (2015) guidelines for prescribing disease-modifying treatments in multiple sclerosis. Pract Neurol. 2015 Aug;15(4):273–279. 26101071 10.1136/practneurol-2015-001139

[11] Norwegian national professional guideline - multiple sclerosis - last professionally updated: 14 September 2022 [2023 Jan 21]. Available from: https://www.helsedirektoratet.no/retningslinjer/multippel-sklerose

[12] Dumitrescu L, Constantinescu CS, Tanasescu R. Siponimod for the treatment of secondary progressive multiple sclerosis. Expert Opin Pharmacother. 2019 Feb;20(2):143–150. 30517042 10.1080/14656566.2018.1551363

[13] Comi G, Hartung HP, Bakshi R, et al. Benefit-Risk Profile of Sphingosine-1-Phosphate Receptor Modulators in Relapsing and Secondary Progressive Multiple Sclerosis. Drugs. 2017 Oct;77(16):1755–1768. 28905255 10.1007/s40265-017-0814-1 PMC5661009

[14] Kappos L, Radue EW, O’Connor P, et al. A placebo-controlled trial of oral fingolimod in relapsing multiple sclerosis. N Engl J Med. 2010 Feb 4;362(5):387–401. 20089952 10.1056/NEJMoa0909494

[15] Feng J, Rensel M. Review of the safety, efficacy and tolerability of fingolimod in the treatment of pediatric patients with relapsing-remitting forms of multiple sclerosis (RRMS). Pediatric Health Med Ther. 2019;10:141–146. 31814792 10.2147/PHMT.S220817 PMC6858833

[16] Chitnis T, Arnold DL, Banwell B, et al. Trial of fingolimod versus interferon beta-1a in pediatric multiple sclerosis. N Engl J Med. 2018 Sep 13;379(11):1017–1027. 30207920 10.1056/NEJMoa1800149

[17] McGinley MP, Cohen JA. Sphingosine 1-phosphate receptor modulators in multiple sclerosis and other conditions. Lancet. 2021 Sep 25;398(10306):1184–1194. 34175020 10.1016/S0140-6736(21)00244-0

[18] Chun J, Giovannoni G, Hunter SF. Sphingosine 1-phosphate receptor modulator therapy for multiple sclerosis: differential downstream receptor signalling and clinical profile effects. Drugs. 2021 Feb;81(2):207–231. 33289881 10.1007/s40265-020-01431-8 PMC7932974

[19] Hla T, Brinkmann V. Sphingosine 1-phosphate (S1P): physiology and the effects of S1P receptor modulation. Neurology. 2011 Feb 22;76(8 Suppl 3):S3–8. 10.1212/WNL.0b013e31820d5ec1 21339489

[20] Coelho RP, Saini HS, Sato-Bigbee C. Sphingosine-1-phosphate and oligodendrocytes: from cell development to the treatment of multiple sclerosis. Prostaglandins Other Lipid Mediat. 2010 Apr;91(3–4):139–144. 19808013 10.1016/j.prostaglandins.2009.04.002

[21] Pan S, Gray NS, Gao W, et al. Discovery of BAF312 (siponimod), a potent and selective S1P receptor modulator. ACS Med Chem Lett. 2013 Mar 14;4(3):333–337. 24900670 10.1021/ml300396r PMC4027137

[22] Bryan L, Kordula T, Spiegel S, et al. Regulation and functions of sphingosine kinases in the brain. Biochim Biophys Acta. 2008 Sep;1781(9):459–466. 18485923 10.1016/j.bbalip.2008.04.008 PMC2712649

[23] Hla T. Physiological and pathological actions of sphingosine 1-phosphate. Semin Cell Dev Biol. 2004 Oct;15(5):513–520. 15271296 10.1016/j.semcdb.2004.05.002

[24] Rosen H, Gonzalez-Cabrera PJ, Sanna MG, et al. Sphingosine 1-phosphate receptor signaling. Annu Rev Biochem. 2009;78:743–768. 19231986 10.1146/annurev.biochem.78.072407.103733

[25] Sanna MG, Liao J, Jo E, et al. Sphingosine 1-phosphate (S1P) receptor subtypes S1P1 and S1P3, respectively, regulate lymphocyte recirculation and heart rate. J Biol Chem. 2004 Apr 2;279(14):13839–13848. 14732717 10.1074/jbc.M311743200

[26] Jenne CN, Enders A, Rivera R, et al. T-bet-dependent S1P5 expression in NK cells promotes egress from lymph nodes and bone marrow. J Exp Med. 2009 Oct 26;206(11):2469–2481. 19808259 10.1084/jem.20090525 PMC2768857

[27] Jaillard C, Harrison S, Stankoff B, et al. Edg8/S1P5: an oligodendroglial receptor with dual function on process retraction and cell survival. J Neurosci. 2005 Feb 9;25(6):1459–1469. 15703400 10.1523/JNEUROSCI.4645-04.2005 PMC6726002

[28] Smith PA, Schmid C, Zurbruegg S, et al. Fingolimod inhibits brain atrophy and promotes brain-derived neurotrophic factor in an animal model of multiple sclerosis. J Neuroimmunol. 2018 May;15(318):103–113. 10.1016/j.jneuroim.2018.02.016 29530550

[29] Okajima F. Plasma lipoproteins behave as carriers of extracellular sphingosine 1-phosphate: is this an atherogenic mediator or an anti-atherogenic mediator? Biochim Biophys Acta. 2002 May 23;1582(1–3):132–137. 12069820 10.1016/s1388-1981(02)00147-6

[30] Brinkmann V, Billich A, Baumruker T, et al. Fingolimod (FTY720): discovery and development of an oral drug to treat multiple sclerosis. Nat Rev Drug Discov. 2010 Nov 9;9(11):883–897. 21031003 10.1038/nrd3248

[31] Kim YM, Sachs T, Asavaroengchai W, et al. Graft-versus-host disease can be separated from graft-versus-lymphoma effects by control of lymphocyte trafficking with FTY720. J Clin Invest. 2003 Mar;111(5):659–669. 12618520 10.1172/JCI16950 PMC151899

[32] McEvoy K, Normile TG, Poeta MD. Antifungal drug development: targeting the fungal sphingolipid pathway. J Fungi (Basel). 2020 Aug 20;6:3. 10.3390/jof6030142 PMC7559796 32825250

[33] Teijaro JR, Walsh KB, Long JP, et al. Protection of ferrets from pulmonary injury due to H1N1 2009 influenza virus infection: immunopathology tractable by sphingosine-1-phosphate 1 receptor agonist therapy. Virology. 2014 Mar;452-453:152–157. 24606692 10.1016/j.virol.2014.01.003 PMC3954990

[34] Resop RS, Fromentin R, Newman D, et al. Fingolimod inhibits multiple stages of the HIV-1 life cycle. PLoS Pathog. 2020 Aug;16(8):e1008679. 32790802 10.1371/journal.ppat.1008679 PMC7425850

[35] Pitman MR, Costabile M, Pitson SM. Recent advances in the development of sphingosine kinase inhibitors. Cell Signal. 2016 Sep;28(9):1349–1363. 27297359 10.1016/j.cellsig.2016.06.007

[36] Gergely P, Nuesslein-Hildesheim B, Guerini D, et al. The selective sphingosine 1-phosphate receptor modulator BAF312 redirects lymphocyte distribution and has species-specific effects on heart rate. Br J Pharmacol. 2012 Nov;167(5):1035–1047. 22646698 10.1111/j.1476-5381.2012.02061.x PMC3485666

[37] Ntranos A, Hall O, Robinson DP, et al. FTY720 impairs CD8 T-cell function independently of the sphingosine-1-phosphate pathway. J Neuroimmunol. 2014 May 15;270(1–2):13–21. 24680062 10.1016/j.jneuroim.2014.03.007

[38] Brown B, Weiss JL, Kolodny S, et al. Analysis of cardiac monitoring and safety data in patients initiating fingolimod treatment in the home or in clinic. BMC Neurol. 2019 Nov 15;19(1):287. 31729968 10.1186/s12883-019-1506-0 PMC6857316

[39] Olsson T, Boster A, Fernandez O, et al. Oral ponesimod in relapsing-remitting multiple sclerosis: a randomised phase II trial. J Neurol Neurosurg Psychiatry. 2014 Nov;85(11):1198–1208. 24659797 10.1136/jnnp-2013-307282 PMC4215282

[40] Noda H, Takeuchi H, Mizuno T, et al. Fingolimod phosphate promotes the neuroprotective effects of microglia. J Neuroimmunol. 2013 Mar 15;256(1–2):13–18. 23290828 10.1016/j.jneuroim.2012.12.005

[41] O’Sullivan C, Schubart A, Mir AK, et al. The dual S1PR1/S1PR5 drug BAF312 (Siponimod) attenuates demyelination in organotypic slice cultures. J Neuroinflammation. 2016 Feb 8;13:31. 26856814 10.1186/s12974-016-0494-x PMC4746808

[42] Kappos L, Bar-Or A, Cree BAC, et al. Siponimod versus placebo in secondary progressive multiple sclerosis (EXPAND): a double-blind, randomised, phase 3 study. Lancet. 2018 Mar 31;391(10127):1263–1273. 29576505 10.1016/S0140-6736(18)30475-6

[43] Tanasescu R, Constantinescu CS. Pharmacokinetic evaluation of fingolimod for the treatment of multiple sclerosis. Expert Opin Drug Metab Toxicol. 2014 Apr;10(4):621–630. 24579791 10.1517/17425255.2014.894019

[44] Calabresi PA, Radue EW, Goodin D, et al. Safety and efficacy of fingolimod in patients with relapsing-remitting multiple sclerosis (FREEDOMS II): a double-blind, randomised, placebo-controlled, phase 3 trial. Lancet Neurol. 2014 Jun;13(6):545–556. 24685276 10.1016/S1474-4422(14)70049-3

[45] Cohen JA, Barkhof F, Comi G, et al. Oral fingolimod or intramuscular interferon for relapsing multiple sclerosis. N Engl J Med. 2010 Feb 4;362(5):402–415. 20089954 10.1056/NEJMoa0907839

[46] Lublin F, Miller DH, Freedman MS, et al. Oral fingolimod in primary progressive multiple sclerosis (INFORMS): a phase 3, randomised, double-blind, placebo-controlled trial. Lancet. 2016 Mar 12;387(10023):1075–1084. 26827074 10.1016/S0140-6736(15)01314-8

[47] De Stefano N, Silva DG, Barnett MH. Effect of fingolimod on brain volume loss in patients with multiple sclerosis. CNS Drugs. 2017 Apr;31(4):289–305. 28247239 10.1007/s40263-017-0415-2 PMC5374177

[48] Brinkmann V, Davis MD, Heise CE, et al. The immune modulator FTY720 targets sphingosine 1-phosphate receptors. J Biol Chem. 2002 Jun 14;277(24):21453–21457. 11967257 10.1074/jbc.C200176200

[49] Kovarik JM, Schmouder R, Barilla D, et al. Single-dose FTY720 pharmacokinetics, food effect, and pharmacological responses in healthy subjects. Br J Clin Pharmacol. 2004 May;57(5):586–591. 15089811 10.1111/j.1365-2125.2003.02065.x PMC1884502

[50] Tugemann B, Manouchehri N, Stuve O. To the editors: impact of mass vaccination on SARS-CoV-2 infections among multiple sclerosis patients taking immunomodulatory disease-modifying therapies in England. Mult Scler Relat Disord. 2022 Jan 19;59:103541. 35078128 10.1016/j.msard.2022.103541 PMC8768009

[51] Tallantyre EC, Vickaryous N, Anderson V, et al. COVID-19 Vaccine Response in People with Multiple Sclerosis. Ann Neurol. 2022 Jan;91(1):89–100. 34687063 10.1002/ana.26251 PMC8652739

[52] Tallantyre EC, Scurr MJ, Vickaryous N, et al. Response to COVID-19 booster vaccinations in seronegative people with MS. medRxiv;2022:2022.03.12.22272083. DOI:10.1016/j.msard.2022.103937. PMC9166227 35700625

[53] Achiron A, Mandel M, Dreyer-Alster S, et al. Humoral immune response to COVID-19 mRNA vaccine in patients with multiple sclerosis treated with high-efficacy disease-modifying therapies. Ther Adv Neurol Disord. 2021;14:17562864211012835. 34035836 10.1177/17562864211012835 PMC8072850

[54] Sormani MP, Inglese M, Schiavetti I, et al. Effect of SARS-CoV-2 mRNA vaccination in MS patients treated with disease modifying therapies. EBioMedicine. 2021 Oct;72:103581. 34563483 10.1016/j.ebiom.2021.103581 PMC8456129

[55] Garjani A, Patel S, Bharkhada D, et al. Impact of mass vaccination on SARS-CoV-2 infections among multiple sclerosis patients taking immunomodulatory disease-modifying therapies in England. Mult Scler Relat Disord. 2022 Jan;57:103458. 34896876 10.1016/j.msard.2021.103458 PMC8645279

[56] Gilenya (fingolimod) Prescribing Information EMA and FDA. [cited 2022 Sep 12]. Available from: http://www.ema.europa.eu/docs/en_GB/document_library/EPAR_-_Product_Information/human/002202/WC500104528.pdf

[57] Karlsson G, Francis G, Koren G, et al. Pregnancy outcomes in the clinical development program of fingolimod in multiple sclerosis. Neurology. 2014 Feb 25;82(8):674–680. 24463630 10.1212/WNL.0000000000000137 PMC3945658

[58] Geissbuhler Y, Vile J, Koren G, et al. Evaluation of pregnancy outcomes in patients with multiple sclerosis after fingolimod exposure. Ther Adv Neurol Disord. 2018;11:1756286418804760. 30542374 10.1177/1756286418804760 PMC6236588

[59] Del Poeta M, Ward BJ, Greenberg B, et al. Cryptococcal meningitis reported with fingolimod treatment: case series. Neurol Neuroimmunol Neuroinflamm. 2022 May;9:3. 10.1212/NXI.0000000000001156 PMC8941596 35318259

[60] Sriwastava S, Kataria S, Srivastava S, et al. Disease-modifying therapies and progressive multifocal leukoencephalopathy in multiple sclerosis: a systematic review and meta-analysis. J Neuroimmunol. 2021 Nov;15(360):577721. 10.1016/j.jneuroim.2021.577721 PMC9810068 34547511

[61] Gold R, Piani-Meier D, Kappos L, et al. Siponimod vs placebo in active secondary progressive multiple sclerosis: a post hoc analysis from the phase 3 EXPAND study. J Neurol. 2022 Sep;269(9):5093–5104. 35639197 10.1007/s00415-022-11166-z PMC9363350

[62] Arnold DL, Piani-Meier D, Bar-Or A, et al. Effect of siponimod on magnetic resonance imaging measures of neurodegeneration and myelination in secondary progressive multiple sclerosis: gray matter atrophy and magnetization transfer ratio analyses from the EXPAND phase 3 trial. Mult Scler. 2022 Sep;28(10):1526–1540. 35261318 10.1177/13524585221076717 PMC9315182

[63] Cree BA, Arnold DL, Fox RJ, et al. Long-term efficacy and safety of siponimod in patients with secondary progressive multiple sclerosis: analysis of EXPAND core and extension data up to >5 years. Mult Scler. 2022;5:13524585221083194. 10.1177/13524585221083194 PMC9315196 35380078

[64] Leavitt VM, Rocca M. Siponimod for cognition in secondary progressive multiple sclerosis: thinking through the evidence. Neurology. 2021 Jan 19;96(3):91–92. 33328322 10.1212/WNL.0000000000011279

[65] Selmaj K, Li DK, Hartung HP, et al. Siponimod for patients with relapsing-remitting multiple sclerosis (BOLD): an adaptive, dose-ranging, randomised, phase 2 study. Lancet Neurol. 2013 Aug;12(8):756–767. 23764350 10.1016/S1474-4422(13)70102-9

[66] Kappos L, Li DK, Stuve O, et al. Safety and efficacy of siponimod (BAF312) in patients with relapsing-remitting multiple sclerosis: dose-blinded, randomized extension of the phase 2 BOLD study. JAMA Neurol. 2016 Sep 1;73(9):1089–1098. 27380540 10.1001/jamaneurol.2016.1451

[67] Jin Y, Borell H, Gardin A, et al. In vitro studies and in silico predictions of fluconazole and CYP2C9 genetic polymorphism impact on siponimod metabolism and pharmacokinetics. Eur J Clin Pharmacol. 2018 Apr;74(4):455–464. 29273968 10.1007/s00228-017-2404-2 PMC5849655

[68] Glaenzel U, Jin Y, Nufer R, et al. Metabolism and disposition of siponimod, a novel selective S1P1/S1P5 agonist, in healthy volunteers and in vitro identification of human cytochrome P450 enzymes involved in its oxidative metabolism. Drug Metab Dispos. 2018 Jul;46(7):1001–1013. 29735753 10.1124/dmd.117.079574

[69] Prescribing Information Mayzent (siponimod) Tablets. [cited 2022 Aug 1]. Available from: https://www.accessdata.fda.gov/drugsatfda_docs/label/2019/209884s000lbl.pdf

[70] Simone IL, Tortorella C, Ghirelli A. Influence of pregnancy in multiple sclerosis and impact of disease-modifying therapies. Front Neurol. 2021;12:697974. 34276545 10.3389/fneur.2021.697974 PMC8280312

[71] Multiple Sclerosis Society. Ozanimod. [cited 1 Aug 2022]. Available from: https://www.mssociety.org.uk/about-ms/treatments-and-therapies/disease-modifying-therapies/ozanimod-zeposia

[72] Cohen JA, Comi G, Selmaj KW, et al. Safety and efficacy of ozanimod versus interferon beta-1a in relapsing multiple sclerosis (RADIANCE): a multicentre, randomised, 24-month, phase 3 trial. Lancet Neurol. 2019 Nov;18(11):1021–1033. 31492652 10.1016/S1474-4422(19)30238-8

[73] Cohen JA, Arnold DL, Comi G, et al. Safety and efficacy of the selective sphingosine 1-phosphate receptor modulator ozanimod in relapsing multiple sclerosis (RADIANCE): a randomised, placebo-controlled, phase 2 trial. Lancet Neurol. 2016 Apr;15(4):373–381. 26879276 10.1016/S1474-4422(16)00018-1

[74] Comi G, Kappos L, Selmaj KW, et al. Safety and efficacy of ozanimod versus interferon beta-1a in relapsing multiple sclerosis (SUNBEAM): a multicentre, randomised, minimum 12-month, phase 3 trial. Lancet Neurol. 2019 Nov;18(11):1009–1020. 31492651 10.1016/S1474-4422(19)30239-X

[75] Koscielny V. Phase III SUNBEAM and RADIANCE PART B trials for Ozanimod in relapsing multiple sclerosis demonstrate superiority versus interferon-beta-1a (Avonex((R))) in reducing annualized relapse rates and MRI brain lesions. Neurodegener Dis Manag. 2018 Jun;8(3):141–142. 29943693 10.2217/nmt-2018-0012

[76] Rasche L, Paul F. Ozanimod for the treatment of relapsing remitting multiple sclerosis. Expert Opin Pharmacother. 2018 Dec;19(18):2073–2086. 30407868 10.1080/14656566.2018.1540592

[77] UK MS Society. Ponesimod [cited 2022 Aug 13]. Available from: https://www.mssociety.org.uk/about-ms/treatments-and-therapies/disease-modifying-therapies/ponesimod-ponvory

[78] Kappos L, Fox RJ, Burcklen M, et al. Ponesimod compared with teriflunomide in patients with relapsing multiple sclerosis in the active-comparator phase 3 OPTIMUM study: a randomized clinical trial. JAMA Neurol. 2021 May 1;78(5):558–567. 33779698 10.1001/jamaneurol.2021.0405 PMC8008435

[79] Tanasescu R, Evangelou N, Constantinescu CS. Role of oral teriflunomide in the management of multiple sclerosis. Neuropsychiatr Dis Treat. 2013;9:539–553. 23637535 10.2147/NDT.S31248 PMC3639219

[80] Baldin E, Lugaresi A. Ponesimod for the treatment of relapsing multiple sclerosis. Expert Opin Pharmacother. 2020 Nov;21(16):1955–1964. 32808832 10.1080/14656566.2020.1799977

[81] Berger JR, Cree BA, Greenberg B, et al. Progressive multifocal leukoencephalopathy after fingolimod treatment. Neurology. 2018 May 15;90(20):e1815–e1821. 29669908 10.1212/WNL.0000000000005529 PMC5957303

[82] Sharma K, Chaudhary D, Beard K, et al. A comprehensive review of varicella-zoster virus, herpes simplex virus and cryptococcal infections associated with sphingosine-1-phosphate receptor modulators in multiple sclerosis patients. Mult Scler Relat Disord. 2022 Feb 8;59:103675. 35168095 10.1016/j.msard.2022.103675

[83] Ufer M, Shakeri-Nejad K, Gardin A, et al. Impact of siponimod on vaccination response in a randomized, placebo-controlled study. Neurol Neuroimmunol Neuroinflamm. 2017 Nov;4(6):e398. 28955715 10.1212/NXI.0000000000000398 PMC5608565

[84] Wolf A, Alvarez E. COVID-19 vaccination in patients with multiple sclerosis on disease-modifying therapy. Neurol Clin Pract. 2021 Aug;11(4):358–361. 34484934 10.1212/CPJ.0000000000001088 PMC8382390

[85] Mallucci G, Zito A, Fabbro BD, et al. Asymptomatic SARS-CoV-2 infection in two patients with multiple sclerosis treated with fingolimod. Mult Scler Relat Disord. 2020 Oct;45:102414. 32711296 10.1016/j.msard.2020.102414 PMC7369008

[86] Garjani APS, Law GR, Bharkhada D, et al. Effectiveness of COVID-19 Vaccines in Multiple Sclerosis Patients Receiving Disease-Modifying Therapies in England. Neurology: Poster presentation S5.008. AAN 2022 Anual Meeting; 2022.

[87] Dumitrescu L, Papathanasiou A, Coclitu C, et al. Beta interferons as immunotherapy in multiple sclerosis: a new outlook on a classic drug during the COVID-19 pandemic. QJM. 2021 Jan 22;114:691–697. 33486513 10.1093/qjmed/hcaa348 PMC7928608

[88] Gombolay GDM, Tyor W. Immune responses to SARS-CoV-2 vaccination in multiple sclerosis: a systematic review and meta-analysis. Poster presentation S5.008. AAN 2022 Anual Meeting2022. 10.1002/acn3.51628 PMC9349877 35852423

[89] Framke E, Pontieri L, Bramow S, et al. Rebound of clinical disease activity after fingolimod discontinuation? A nationwide cohort study of patients in Denmark. J Neurol Neurosurg Psychiatry. 2022 Sep;28. 10.1136/jnnp-2022-329607 36171103

[90] Legangneux E, Gardin A, Johns D. Dose titration of BAF312 attenuates the initial heart rate reducing effect in healthy subjects. Br J Clin Pharmacol. 2013 Mar;75(3):831–841. 22845008 10.1111/j.1365-2125.2012.04400.x PMC3575950

[91] van der Weyden L, Arends MJ, Campbell AD, et al. Genome-wide in vivo screen identifies novel host regulators of metastatic colonization. Nature. 2017 Jan 12;541(7636):233–236. 28052056 10.1038/nature20792 PMC5603286

[92] Cantalupo A, Gargiulo A, Dautaj E, et al. S1PR1 (sphingosine-1-phosphate receptor 1) signaling regulates blood flow and pressure. Hypertension. 2017 Aug;70(2):426–434. 28607130 10.1161/HYPERTENSIONAHA.117.09088 PMC5531041

[93] Stamatellos VP, Rigas A, Stamoula E, et al. S1P receptor modulators in multiple sclerosis: detecting a potential skin cancer safety signal. Mult Scler Relat Disord. 2022 Mar;59:103681. 35168096 10.1016/j.msard.2022.103681

[94] Hellmann MA, Lev N, Lotan I, et al. Tumefactive demyelination and a malignant course in an MS patient during and following fingolimod therapy. J Neurol Sci. 2014 Sep 15;344(1–2):193–197. 25001515 10.1016/j.jns.2014.06.013

[95] Hussain R, O’Leary S, Pacheco FM, et al. Acute relapse after initiation of Siponimod in a patient with secondary progressive MS. J Neurol. 2016 Mar;263(3):606–610. 26914924 10.1007/s00415-015-7999-6

[96] Fujii C, Kondo T, Ochi H, et al. Altered T cell phenotypes associated with clinical relapse of multiple sclerosis patients receiving fingolimod therapy. Sci Rep. 2016 Oct;18(6):35314. 10.1038/srep35314 PMC5082790 27752051

[97] Giordana MT, Cavalla P, Uccelli A, et al. Overexpression of sphingosine-1-phosphate receptors on reactive astrocytes drives neuropathology of multiple sclerosis rebound after fingolimod discontinuation. Mult Scler. 2018 Jul;24(8):1133–1137. 29708466 10.1177/1352458518763095

[98] Fox E, Edwards K, Burch G, et al. Outcomes of switching directly to oral fingolimod from injectable therapies: results of the randomized, open-label, multicenter, evaluate patient outcomes (EPOC) study in relapsing multiple sclerosis. Mult Scler Relat Disord. 2014 Sep;3(5):607–619. 26265273 10.1016/j.msard.2014.06.005

[99] Benedict RHB, Tomic D, Cree BA, et al. Siponimod and cognition in secondary progressive multiple sclerosis: EXPAND secondary analyses. Neurology. 2021 Jan 19;96(3):e376–e386. 33328324 10.1212/WNL.0000000000011275

[100] Spampinato SF, Merlo S, Sano Y, et al. Protective effect of the sphingosine-1 phosphate receptor agonist siponimod on disrupted blood brain barrier function. Biochem Pharmacol. 2021 Apr;186:114465. 33577891 10.1016/j.bcp.2021.114465

[101] Colombo E, Bassani C, De Angelis A, et al. Siponimod (BAF312) activates Nrf2 while hampering nfkappab in human astrocytes, and protects from astrocyte-induced neurodegeneration. Front Immunol. 2020;11:635. 32322257 10.3389/fimmu.2020.00635 PMC7156595

[102] Goncuoglu C, Tuncer A, Bayraktar-Ekincioglu A, et al. Factors associated with fingolimod rebound: a single center real-life experience. Mult Scler Relat Disord. 2021 Nov;56:103278. 34655957 10.1016/j.msard.2021.103278

[103] Barboza A, Gaitan MI, Alonso R, et al. Rebound activity after fingolimod cessation: a case - control study. Mult Scler Relat Disord. 2022 Jan;57:103329. 35158443 10.1016/j.msard.2021.103329

[104] Sullivan R, Kilaru A, Hemmer B, et al. COVID-19 infection in fingolimod- or siponimod-treated patients: case series. Neurol Neuroimmunol Neuroinflamm. 2021 Nov;9(1). doi:10.1212/NXI.0000000000001092. PMC8860466 34848501

